# Identification of therapeutic targets and prognostic biomarkers in the Siglec family of genes in tumor immune microenvironment of sarcoma

**DOI:** 10.1038/s41598-023-50758-1

**Published:** 2024-01-05

**Authors:** Lili Qi, Kuiying Jiang, Fei-fei Zhao, Ping Ren, Ling Wang

**Affiliations:** 1 Department of Orthopedic Oncology, The Third Hospital of Hebei Medical University, Shijiazhuang, Hebei, People’s Republic of China; 2 Department of Orthopedic Research Center, The Third Hospital of Hebei Medical University, Shijiazhuang, People’s Republic of China; 3 Experimental Center for Teaching of Hebei Medical University, Shijiazhuang, Hebei, People’s Republic of China; 4 National Demonstration Center for Experimental Basic Medical Education, Capital Medical University, Beijing, People’s Republic of China; 5 Department of Orthopedics, The Fourth Hospital of Hebei Medical University, Shijiazhuang, Hebei, People’s Republic of China

**Keywords:** Cell biology, Computational biology and bioinformatics, Immunology, Oncology

## Abstract

Sarcomas (SARC) are a highly heterogeneous cancer type that is prone to recurrence and metastasis. Numerous studies have confirmed that Siglecs are involved in immune signaling and play a key role in regulating immune responses in inflammatory diseases and various cancers. However, studies that systematically explore the therapeutic and prognostic value of Siglecs in SARC patients are very limited. The online databases GEPIA, UALCAN, TIMER, The Kaplan–Meier Plotter, GeneMANIA, cBioPortal, and STING were used in this study. IHC staining was performed on the collected patient tissues, and clinical data were statistically analyzed. The transcript levels of most Siglec family members showed a high expression pattern in SARC. Compared with normal tissues, *Siglec-5*, *Siglec-10*, and *Siglec-12* were abnormally highly expressed in tumor tissues. Importantly, *Siglec-15* was significantly associated with poor prognosis. Functional enrichment analysis showed that the Siglec family was mainly enriched in hematopoietic cell lineages. The genes associated with molecular mutations in the Siglec family were mainly TP53 and MUC16, among which *Siglec-2* and *Siglec-15* were significantly associated with the survival of patients. The expression levels of all Siglec family members were significantly correlated with various types of immune cells (B cells, CD8 + T cells, CD4 + T cells, macrophages, neutrophils and dendritic cells). Furthermore, a significant correlation was found between the somatic copy number changes of all Siglec molecules and the abundance of immune infiltrates. Our study paints a promising vision for the development of immunotherapy drugs and the construction of prognostic stratification models by investigating the therapeutic and prognostic potential of the Siglec family for SARC.

## Introduction

SARC arise from mesodermal tissue and are considered to be heterogeneous malignancies. The overall incidence of SARC is less than 5/10 million per year, accounting for less than 1% of all cancers; therefore, it is known as the "forgotten cancer^1^". Among them, osteosarcoma, chondrosarcoma and Ewing sarcoma are the most common bone malignancies^2,3^. According to previous reports, the 5-year survival rate of sarcoma is merely 60–70%^4^, and the treatment of sarcoma remains challenging.

The Sarcoma Foundation of America (SFA) has reported that approximately 20% of sarcoma cases can be cured with surgery, and another 30% can be effectively treated with surgery, chemotherapy, and/or radiation. Therefore, for the treatment of sarcoma, multimodal treatment methods are generally recommended, including surgery, radiotherapy, chemotherapy and immunotherapy^5,6^. At present, a variety of monoclonal antibodies have been developed and applied to target immune checkpoints, such as anti-cytotoxic T lymphocyte antigen 4 (CTLA-4), B7-H3, programmed death receptor 1 (PD-1) and its ligand (PD-L1). But most checkpoint inhibitors are ineffective in clinical treatment, including sarcoma^7,8^. In the case of patients with osteosarcoma, although the 5-year event-free survival (EFS) rate for non-metastatic patients has improved from 17 to 70%, the overall 10-year survival rate for metastatic patients remains below 20%^9,10^. Therefore, the search for new immune checkpoints is imminent.

Siglecs are a class of classical immunoglobulin-like lectins, which are expressed on the surface of myeloid cells and immune cells and can specifically recognize sialic acid^11^. Currently, there are 15 kinds of human Siglec molecules, which are usually divided into two categories: Siglecs with conserved sequences, including *Siglec-1* (sialoadhesin, CD169), *Siglec-2* (CD22), *Siglec-4* (myelin-associated glycoprotein, MAG), and *Siglec-15*, and Siglecs with variable sequences related to CD33, including *Siglec-3*, *5*,* 6*,* 7*,* 8*,* 9*, *10*, *11*, *14*, and *16*^12^. Accumulating evidence suggests that Siglecs are involved in immune signaling and play a pivotal role in regulating immune responses in inflammatory diseases and a variety of cancers^13–16^.

Previous studies have found that several Siglec family members are involved in the occurrence and progression of sarcoma^17^. *Siglec-1* is highly expressed on macrophages present in AIDS-related Kaposi's sarcoma lesions^18,19^. *Siglec-3* is positive in 86% of head and neck myeloid sarcoma samples evaluated^20^. *Siglec-4* can increase the aggressiveness of Ewing's sarcoma through an angiogenesis-mimicking process^21^. *Siglec-15* has been shown to be expressed in osteosarcoma cells and may inhibit proliferation in osteosarcoma through the signal transducer and activator of transcription 3 (STAT3/Bcl-2) pathway, while inducing apoptosis and pyroptosis^22^. However, the expression and mechanism of action of other family molecules in sarcoma remain unknown. Therefore, the purpose of our research is to summarize the clinical research value of Siglecs in SARC. By applying public data to the analysis of its expression, prognostic impact, gene mutation and the correlation with immune infiltration level, we may find effective markers for clinical prediction and treatment of SARC.

## Results

### Differential expression of siglec family members in SARC

**Figure 1:**
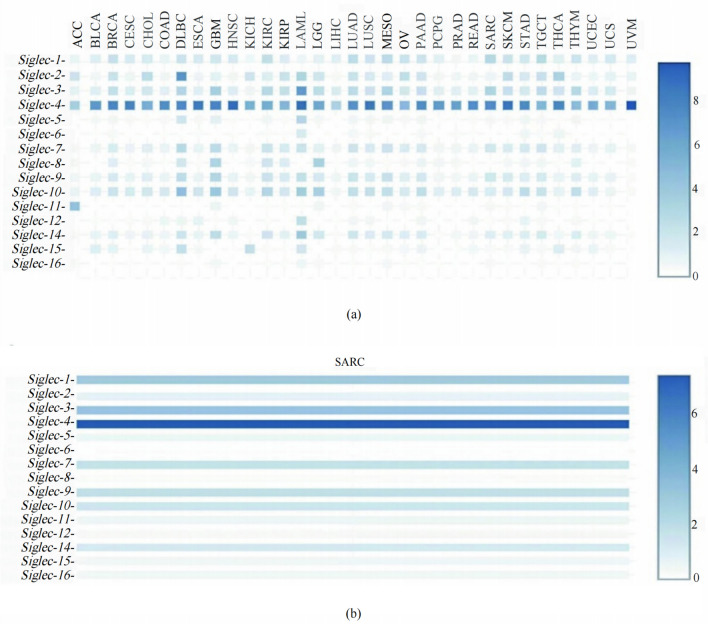
The mRNA expression of Siglecs analyzed by GEPIA. (**a**) The mRNA expression of Siglecs in different types of tumors. (**b**) The mRNA expression of Siglecs in SARC. The relative expression of *Siglec-4* was the highest scored at 7.3 while *Siglec-6* scored 0.1 as the lowest counterpart. ACC: adrenocortical carcinoma, BLCA: bladder urothelial carcinoma, BRC: breast invasive carcinoma, CESC: cervical squamous cell carcinoma and endocervicaladenocarcinoma, CHOL: cholangiocarcinoma, COAD: colon adenocarcinoma, DLBC: lymphoid neoplasm diffuse large b-cell lymphoma, ESCA: esophageal carcinoma, GBM: glioblastoma multiforme, HNSC: head and neck squamous cell carcinoma, KICH: kidney chromophobe, KIRC: kidney renal clear cell carcinoma, KIRP: kidney renal papillary cell carcinoma, LAML: acute myeloid leukemia, LGG: brain lower grade glioma, LIHC: liver hepatocellular carcinoma, LUAD: lung adenocarcinoma, LUSC: lung squamous cell carcinoma, MESO: Mesothelioma, OV: Ovarian serous cystadenocarcinoma, PAAD: pancreatic adenocarcinoma, PCPG: pheochromocytoma and paraganglioma, PRAD: prostate adenocarcinoma, READ: rectum adenocarcinoma, SARC: sarcoma, SKCM: skin cutaneous melanoma, STAD: stomach adenocarcinoma, TGCT: testicular germ cell tumors, THCA: thyroid carcinoma, THYM: thymoma, UCEC: uterine corpus endometrial carcinoma, UCS: uterine carcinosarcoma, UVM: uveal melanoma.

Through the GEPIA database, we first compared the mRNA expression of Siglecs in different types of tumors (Fig. 1a). The result showed that Siglec family members were differentially expressed in cancers. Next, the comprehensive expression of each molecule in the SARC tumor samples was examined. As shown in Fig. 1b, *Siglec-4* had the highest relative expression level with a score of 7.3, while *Siglec-6* had the lowest score of 0.1.

**Figure 2:**
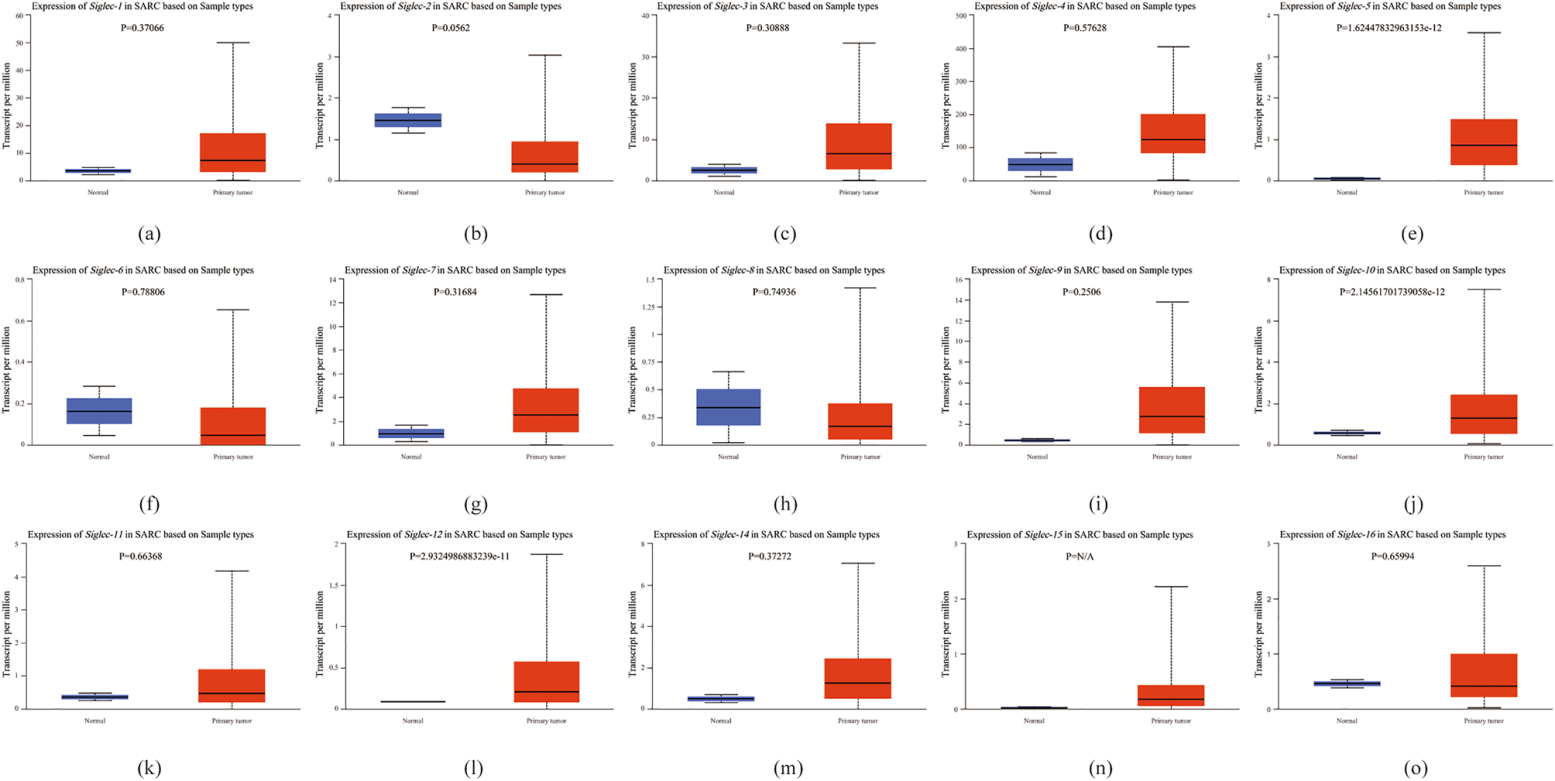
The mRNA expression of Siglecs in SARC tissue and normal tissue analyzed by UALCAN. *Siglec-5*, *Siglec-10*, *Siglec-12* (**e**, **j**, **l**) showed a significantly high expression pattern in SARC regarding normal tissue analyzed quantitatively at the RNA level. Red color means SARC tissues and blue color means normal tissues. The p value thresholds were set at 0.05.

**Figure 3:**
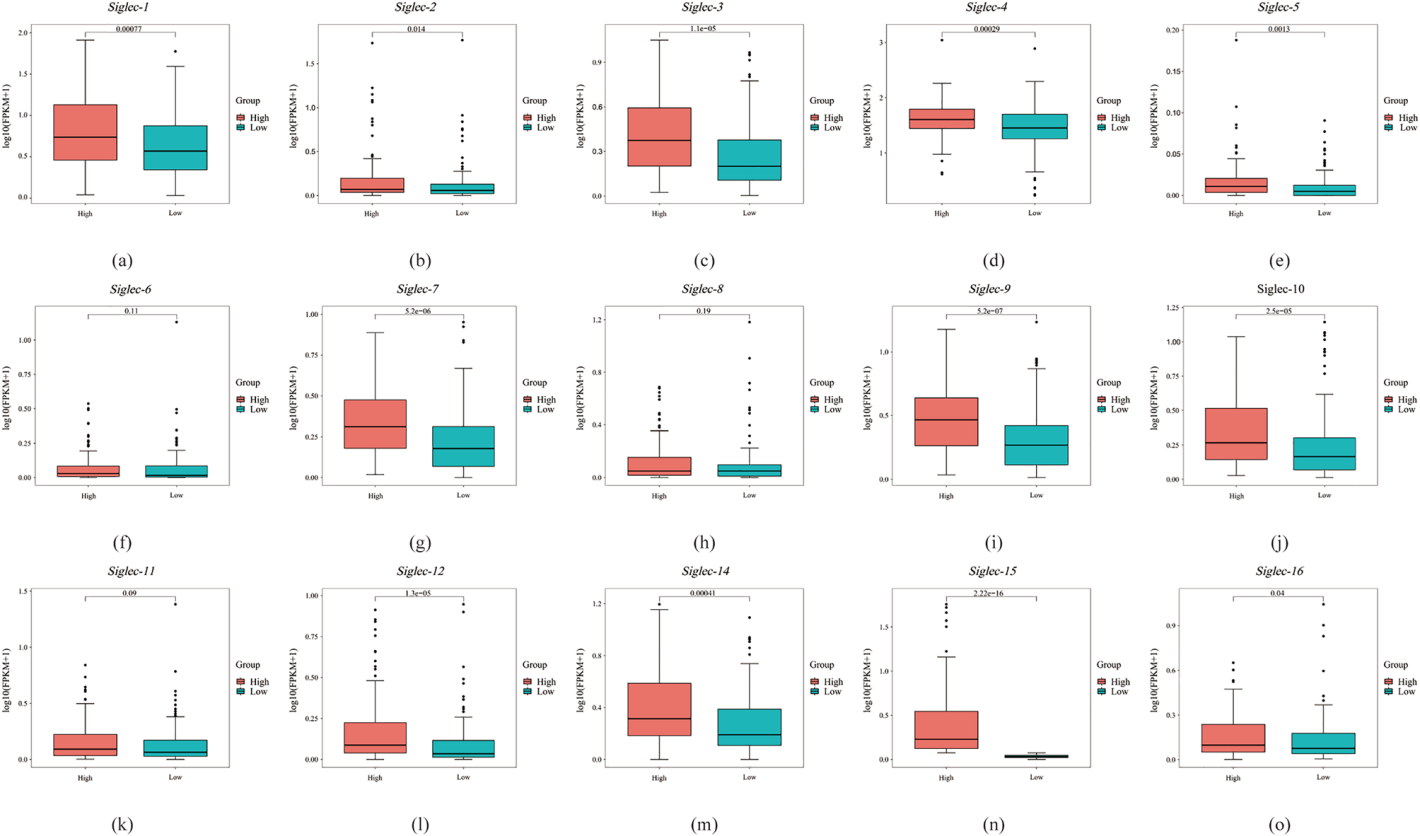
Differential expression of Siglec family molecules between different expression groups in SARC. (**a**) *Siglec-1*, (**b**) *Siglec-2*, (**c**) *Siglec-3*, (**d**) *Siglec-5*, (**e**) *Siglec-6*, (**f**) *Siglec-7*, (**g**) *Siglec-8*, (**h**) *Siglec-9*, (**i**) *Siglec-10*, (**j**) *Siglec-11*, (**k**) *Siglec-12*, (**l**) *Siglec-14*, (**m**) *Siglec-15*, (**n**) *Siglec-16*.

In addition, we compared 260 tumor samples with 2 normal tissue samples to detect the expression of all molecules in the Siglec family. The results showed that the expression levels of *Siglec-5*, *Siglec-10*, and *Siglec-12* were significantly up-regulated (*p* < 0.05) in SARC, whereas the expressions of other counterpart Siglecs genes exhibited insignificant difference (Fig. 2a–o). Next, we downloaded the gene sequencing data of SARC samples in TCGA database, grouped them into low expression group and high expression group according to the median expression of each gene in the samples, and then analyzed whether there was any difference between groups. The results showed that except for *Siglec-6* (*p* = 0.11), *Siglec-8*(p = 0.19) and *Siglec-11* (*p* = 0.09), there were differences among other Siglec family members (Fig. 3).

**Figure 4:**
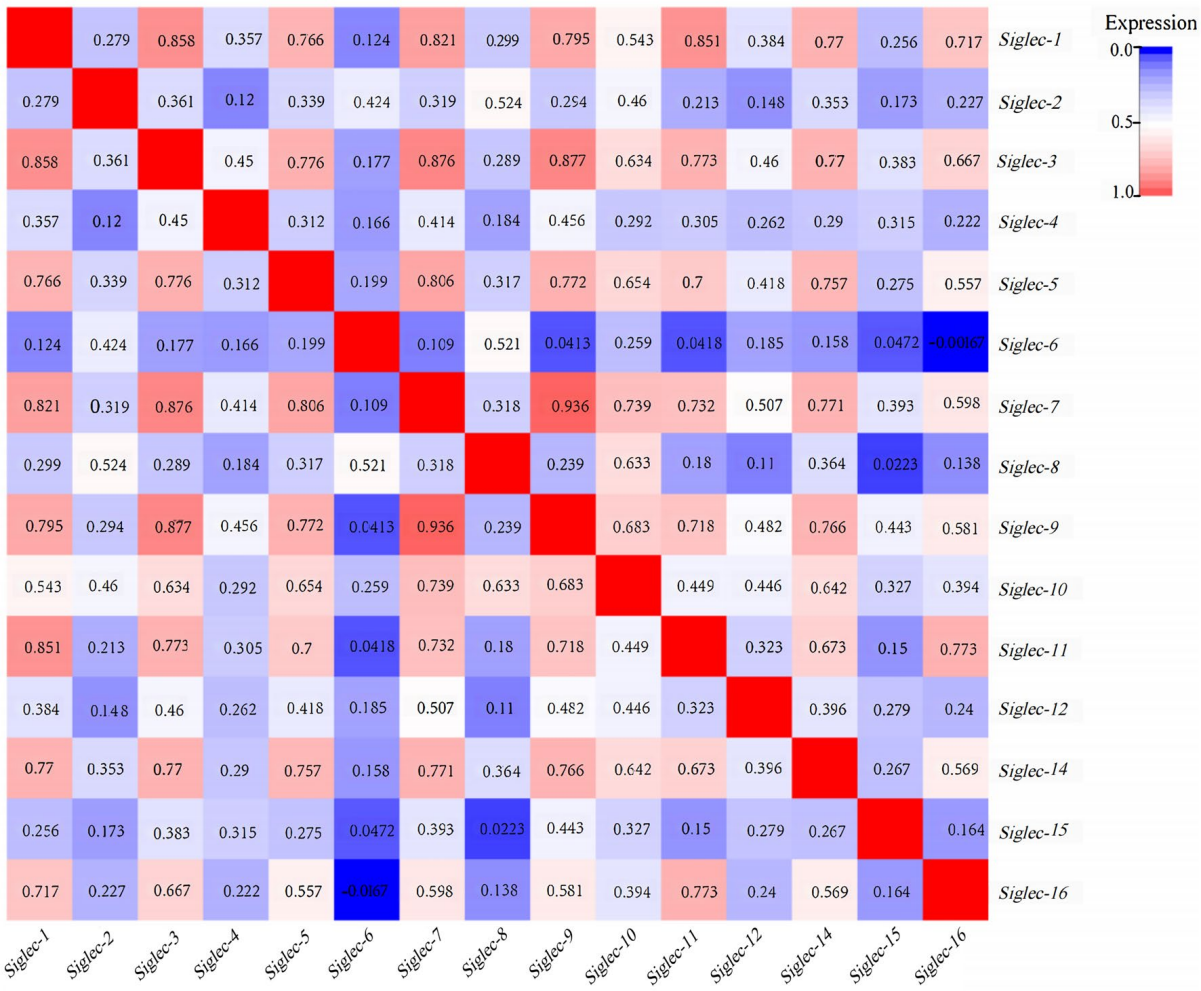
Correlation of differentially expressed molecules of Siglec family at the RNA level.

We also assessed the correlation of differentially expressed molecules at the RNA level. Co-expression analysis revealed moderate correlations between *Siglec-1* and *Siglec-3*, between *Siglec-1* and *Siglec-11*, between *Siglec-3* and *Siglec-7*, between *Siglec-3* and *Siglec-9*, between *Siglec-7* and *Siglec-9*, and between *Siglec-5* and *Siglec-7* (Fig. 4).

### Prognostic value of the siglec family in patients with SARC

**Figure 5:**
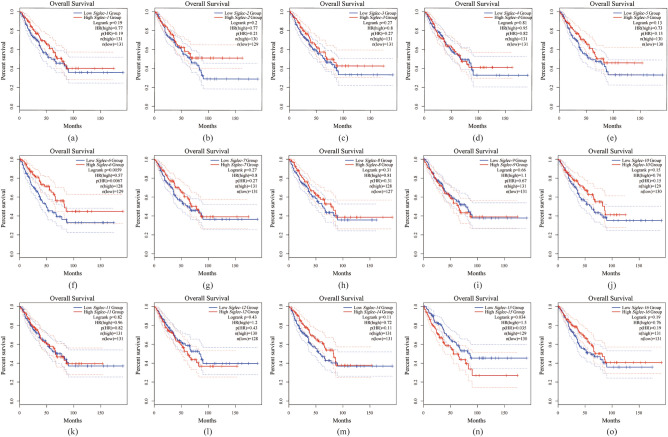
Kaplan–Meier survival curves of OS comparing the high and low expression of Siglec family in SARC. (**a**) *Siglec-1*, (**b**) *Siglec-2*, (**c**) *Siglec-3*, (**d**) *Siglec-5*, (**e**) *Siglec-6*, (**f**) *Siglec-7*, (**g**) *Siglec-8*, (**h**) *Siglec-9*, (**i**) *Siglec-10*, (**j**) *Siglec-11*, (**k**) *Siglec-12*, (**l**) *Siglec-14*, (**m**) *Siglec-15*, (**n**) *Siglec-16*. The log-rank *p* value thresholds were set at 0.05. OS: overall survival.

To further elucidate the prognostic value of Siglec family members in patients with SARC, we downloaded clinical data of patients with SARC from the TCGA database and divided the molecules into high-expression and low-expression groups according to the median expression of each gene in the samples, survival analysis was then performed. The analysis found only between-group differences in *Siglec-6* (*p* = 0.0067) and *Siglec-15* (*p* = 0.035). Among them, patients with low expression of *Siglec-6* have a poor prognosis in patients with SARC, while high expression of *Siglec-15* is associated with a poor prognosis in patients with SARC (Fig. 5a–o).

### Siglec family related gene mutation, expression and interaction analysis

**Figure 6:**
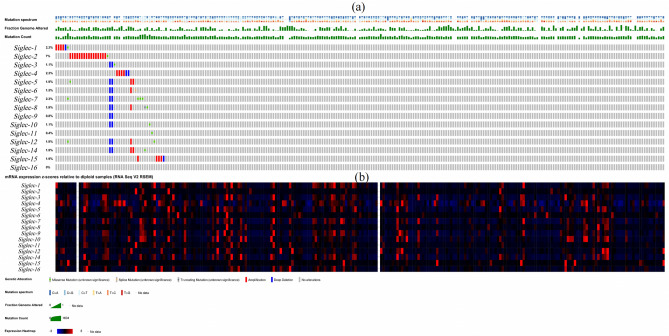
Systemic analysis of genetic alteration (cBioPortal). (**a**) Waterfall map of alterations in different expressed Siglecs in SARC. (**b**) Heat map of alterations in different expressed Siglecs in SARC.

We extracted genomic alterations for each Siglecs gene and RNA-Seq by Expectation–Maximization (RSEM) values in the SARC-TCGA cohort using quantification data from RNA-seq available with the cBioPortal tool. The frequency and types of mutations in genes are shown in waterfall plots and heat maps (Fig. 6). We found that 47 of the 265 samples had genetic mutations, with the mutation ratio of 18%. Among them, *Siglec-2* had the highest mutation rate (7%), and *Siglec-1*, *4*, and *7* ranked second (2.3%), followed by *Siglec-5*, *8*, *12*, *14*, and *15* (1.9%).

**Figure 7:**
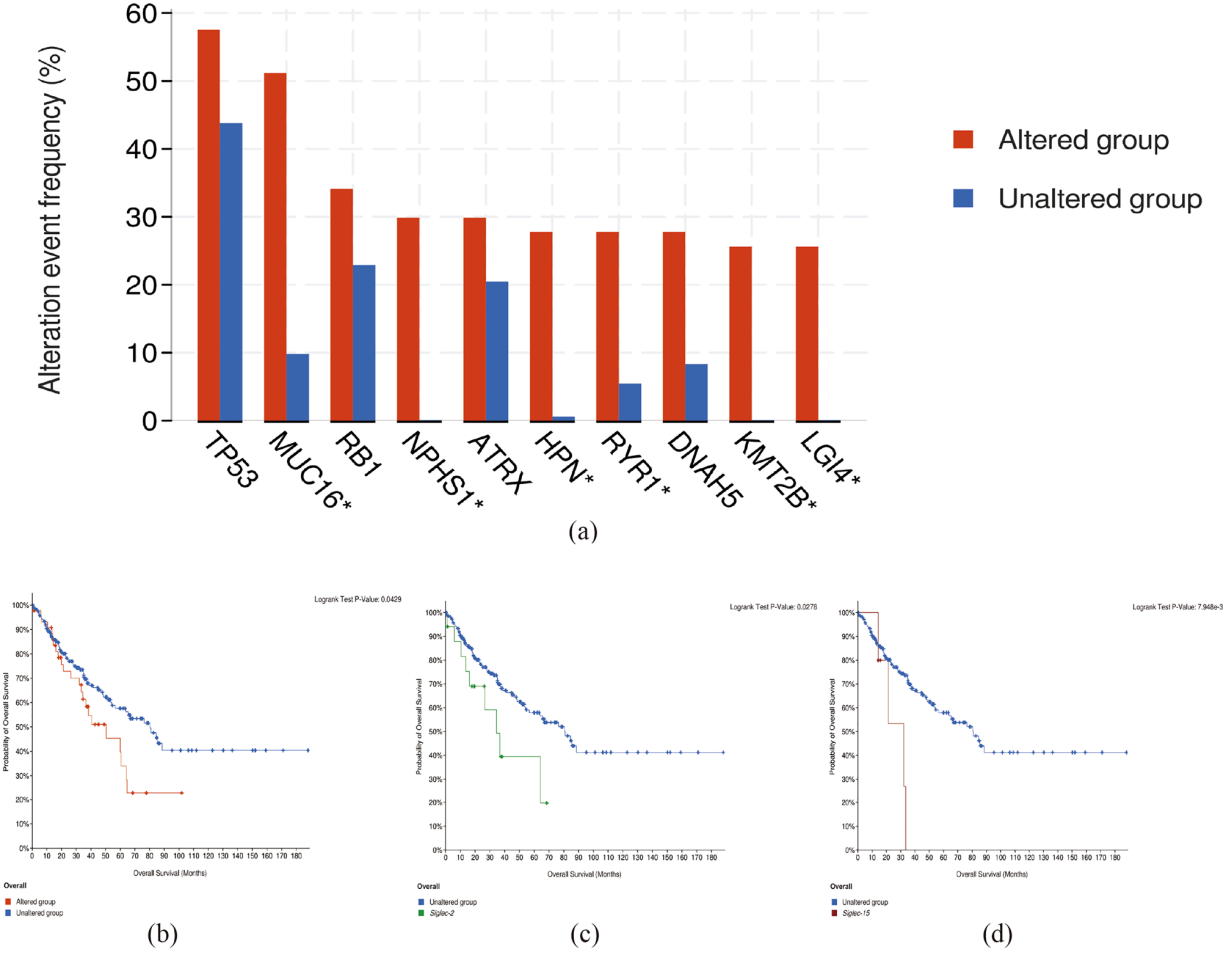
Histogram of genes with the highest frequency in the genome and Siglecs survival curve(cBioPortal). (**a**) Histogram of genes with the highest frequency in the genome. (**b**) Overall survival curve of Siglec family. (**c**) *Siglec-2* overall survival curve. (**d**) *Siglec-15* overall survival curve.

The analysis found that tumor protein (TP53) and Mucin16 (MUC16) had a higher mutation frequency in the Siglec family mutation group (Fig. 7a). Next, we performed survival analysis on the altered group and the unaltered group. There was a significantly difference between the altered group and the unaltered group (*p* = 0.0429) (Fig. 7b). Among them, the OS rate was lower in the mutant *Siglec-2* (*p* = 0.0278) and *Siglec-15* (*p* = 7.948e-3) group than in the unchanged group (Fig. 7c,d), suggesting that the mutant *Siglec-2* and *Siglec-15* were associated with poorer prognosis in sarcoma patients.

**Figure 8:**
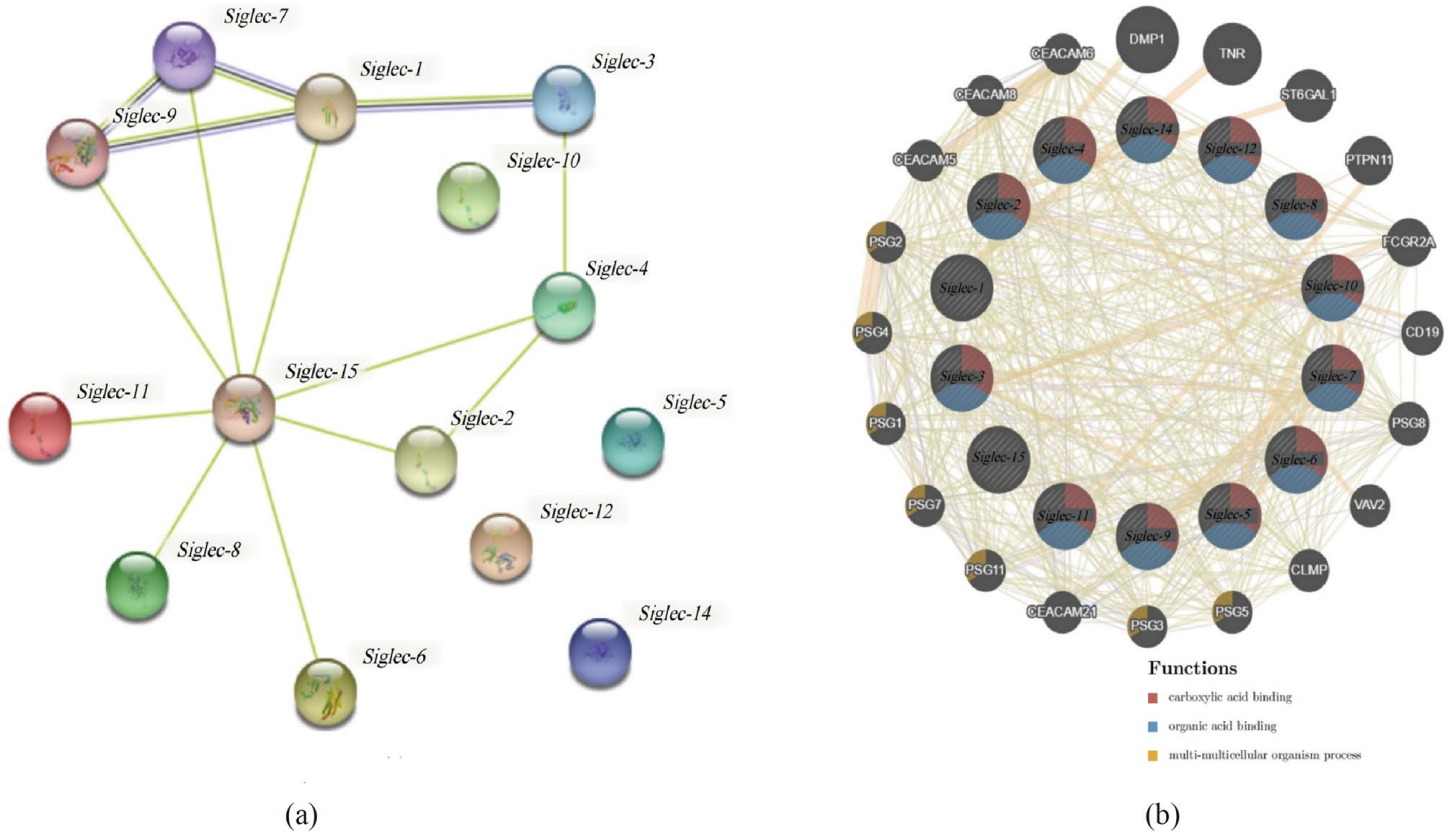
Protein–protein mutual aid and interaction analysis diagram of Siglecs. (**a**) Protein protein mutual aid analysis diagram of Siglecs (String). (**b**) Proteinprotein interaction analysis diagram of Siglecs and its related molecules (GeneMANIA).

The Protein–Protein Interaction Networks (PPI-Net) was constructed to investigate the interactions of the Siglec family molecules at the protein level on the basis of data in the STRING protein query (Fig. 8a). The results showed that the PPI-Net had 14 nodes and 14 edges with an average node degree of 2. The average local clustering coefficient was 0.498. The expected number of edges was 0, and the PPI-Net enrichment p-value was less than 1.0e-16 (Table 1). Furthermore, KEGG pathway analysis obtained from the STRING database also revealed enrichment for hematopoietic cell lineages (Table 2). In addition, we used the GeneMANIA web tool to analyze Siglecs again. The analysis indicated that Siglecs and related molecules such as DMP1, TNR, ST6GAL1, and the functions of differentially expressed genes were mainly involved in some biological processes such as carboxylic acid binding, organic acid binding, multi-multicellular organism process, secretory granule membrane and tertiary granule (Fig. 8b).

**Table 1:** Network Stats of Siglecs.

Number of nodes: 14	Avg.local clustering coefficient: 0.498
Number of edges: 14	Excepted number of edges: 0
Average node degree: 2	PPI enrichment *p* value: < 1.0e − 16

**Table 2:** KEGG Pathway enrichment of Siglecs.

Description	Count in network	Strength	False discovery rate
Hematopoietic cell lineage	3 of 9	1.66	0.0126

### Analysis of siglecs-related immune infiltration in SARC

**Figure 9:**
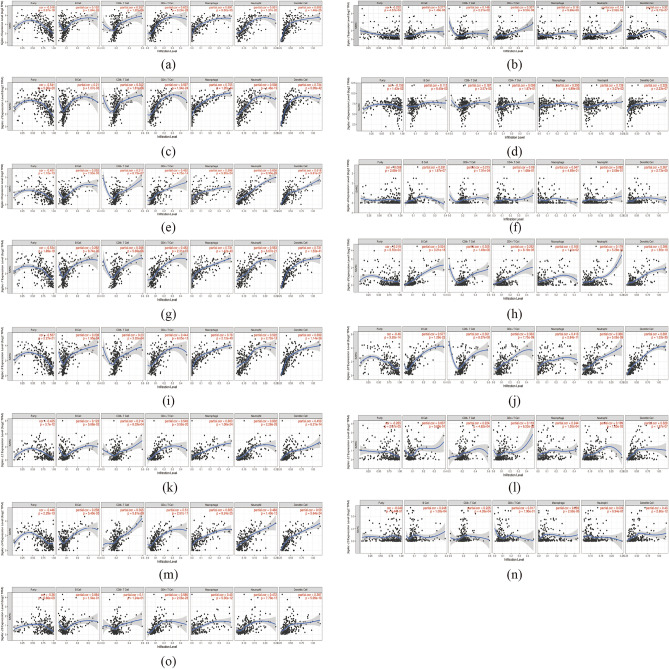
Correlation analysis of interested Siglec family and immune cell infiltration (TIMER). The correlation within immune cell infiltration and transcriptional expression of (**a**) *Siglec-1*, (**b**) *Siglec-2*, (**c**) *Siglec-3*, (**d**) *Siglec-4*, (**e**) *Siglec-5*, (**f**) *Siglec-6*, (**g**) *Siglec-7*, (**h**) *Siglec-8*, (**i**) *Siglec-9*, (**j**) *Siglec-10*, (**k**) *Siglec-11*, (**l**) *Siglec-12*, (**m**) *Siglec-14*, (**n**) *Siglec-15*, and (**o**)*Siglec-16* in SARC cases. SARC: sarcoma.

**Figure 10:**
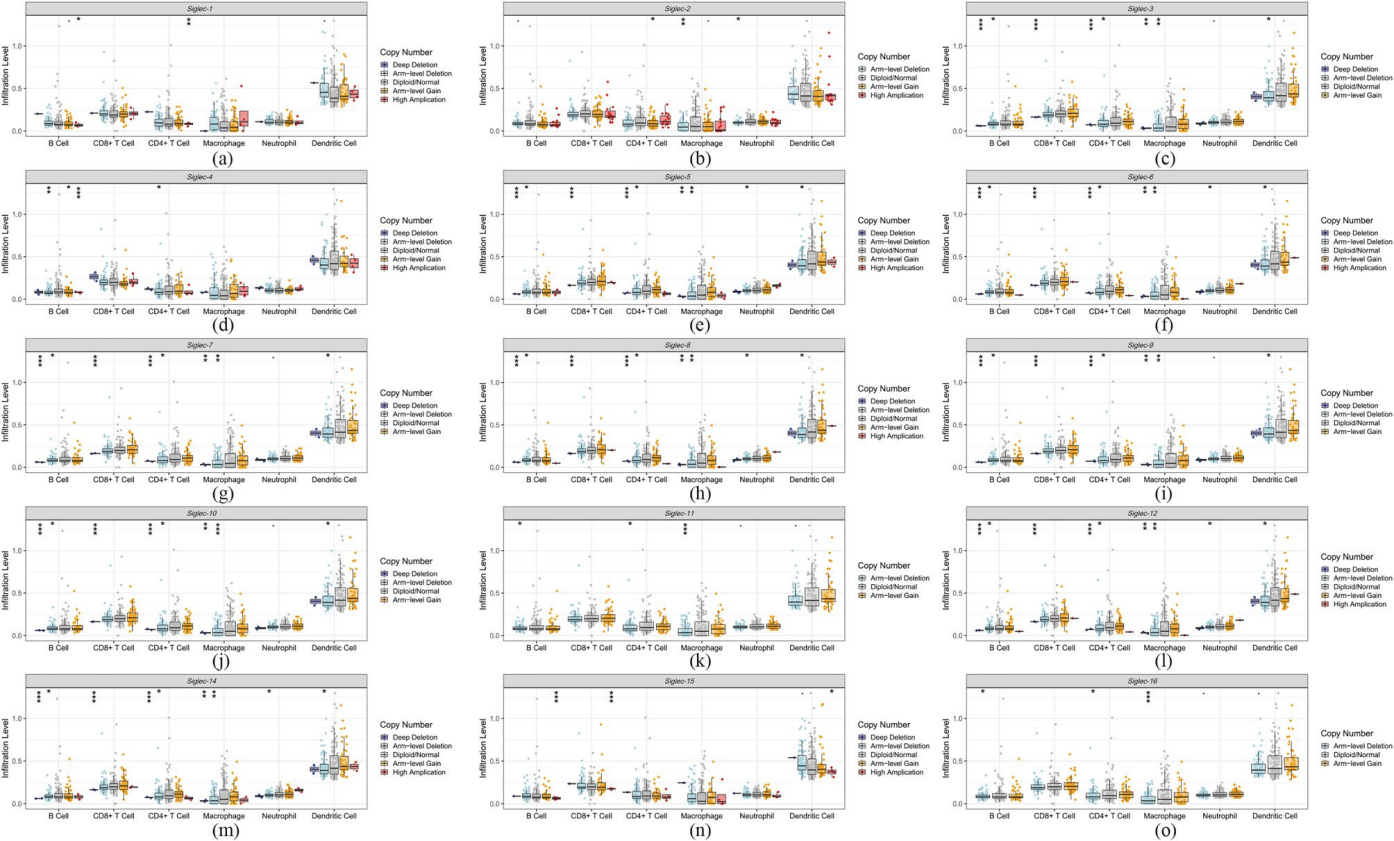
Correlation analysis of SCNA of interested Siglec family and immune infiltration (TIMER). The correlation within immune cell infiltration and SCNA of (**a**) *Siglec-1*, (**b**) *Siglec-2Siglec-2*, (**c**) *Siglec-3*, (**d**) *Siglec-4*, (**e**) *Siglec-5*, (**f**) *Siglec-6*, (**g**) *Siglec-7*, (**h**) *Siglec-8*, (**i**) *Siglec-9*, (**j**) *Siglec-10*, (**k**) *Siglec-11*, (**l**) *Siglec-12*, (**m**) *Siglec-14*, (**n**) *Siglec-15*, and (**o**) *Siglec-16* in SARC cases. *P < 0.05, **P < 0.01, ***P < 0.001. SCNA: somatic copy number alterations.

Evidence has shown that Siglecs were not only abnormally expressed on the tumor cells, but on the other types of infiltrating immune cells, which revealed the importance of these molecules as potential targets for immune therapy. Considering this, the correlations between the Siglec family and immune cell infiltration in SARC were determined by TIMER (Table 3 and Fig. 9). The purity-corrected partial Spearman’s rho value and statistical significance were shown by the scatterplots. We found that the expression levels of *Siglec-1*,* 2*, *3*, *5*, *7*,* 8*, *9*, *10*, and *14* were significantly (*p* < 0.05) and positively associated with all types of immune cells including B cells, CD8 + T cells, CD4 + T cells, macrophages, neutrophils, and dendritic cells. In addition, *Siglec-4* and *Siglec-12* were both positively associated with CD8 + T cells, macrophages, neutrophils, and dendritic cells. *Siglec-6* expression was shown to have a positive correlation with B cells, CD8 + T cells and dendritic cells infiltration. The correlation between B cells and *Siglec-11* was not statistically significant. *Siglec-15* was positively associated with B cells, CD8 + T cells, macrophages, and dendritic cells, whereas *Siglec-16* was positively associated with CD4 + T cells, macrophages, neutrophils, and dendritic cells. Next, we analyzed the correlation between somatic copy number alterations (SCNA) and abundance of immune infiltrates of Siglec family (Fig. 10). The results showed that the deep deletion of Siglec family members except *Siglec-1*, *2*, *4*, *16*, the arm-level deletion of Siglec family members except *Siglec-1*, *2*, *11*, the arm-level gain and high amplication of *Siglec-4* were significantly correlated with the B cell infiltration level. The deep deletion of *Siglec-3*,* 5*, *6*, *7*,* 8*, *9*, *10*, *12*, *14*, and *15* was significantly correlated with the CD8 + T cell infiltration level. The deep deletion of *Siglec-3*, *5*, *6*, *7*, *8*, *9*, *10*, *12*, *13*, and *14*, the arm-level deletion of *Siglec-3*, *15*, high amplication of *Siglec-1* and arm-level gain of *Siglec-2* were significantly correlated with CD4 + T cells. The arm-level deletion of Siglec family members was significantly correlated with dendritic cells, except for the expressions of *Siglec-1*, *2*, *4*, and *11*. The arm-level deletion of Siglec family members was significantly correlated with the macrophage infiltration level, except for the expressions of *Siglec-1* and *Siglec-4*. The arm-level deletion of *Siglec-2*, *5*, *6*, *8*, *12*, *14*, and *15* was significantly correlated with the neutrophil cell infiltration level.

**Table 3:** Correlations between Siglecs expression and immune cell infiltration.

	B cell	CD8^+^T cell	CD4^+^T cell	Macrophage	Neutrophil	Dendritic cell
*Siglec-1*	P	P	P	P	P	P
*Siglec-2*	P	P	P	P	P	P
*Siglec-3*	P	P	P	P	P	P
*Siglec-4*	O	P	O	P	P	P
*Siglec-5*	P	P	P	P	P	P
*Siglec-6*	P	P	O	O	O	P
*Siglec-7*	P	P	P	P	P	P
*Siglec-8*	P	P	P	P	P	P
*Siglec-9*	P	P	P	P	P	P
*Siglec-10*	P	P	P	P	P	P
*Siglec-11*	O	P	P	P	P	P
*Siglec-12*	O	P	O	P	P	P
*Siglec-14*	P	P	P	P	P	P
*Siglec-15*	P	P	O	P	O	P
*Siglec-16*	O	O	P	P	P	P

*P* positive; *N* negative; *O* without correlation; *Siglec* sialic acid-binding immunoglobulin-like lectin.

**Figure 11:**
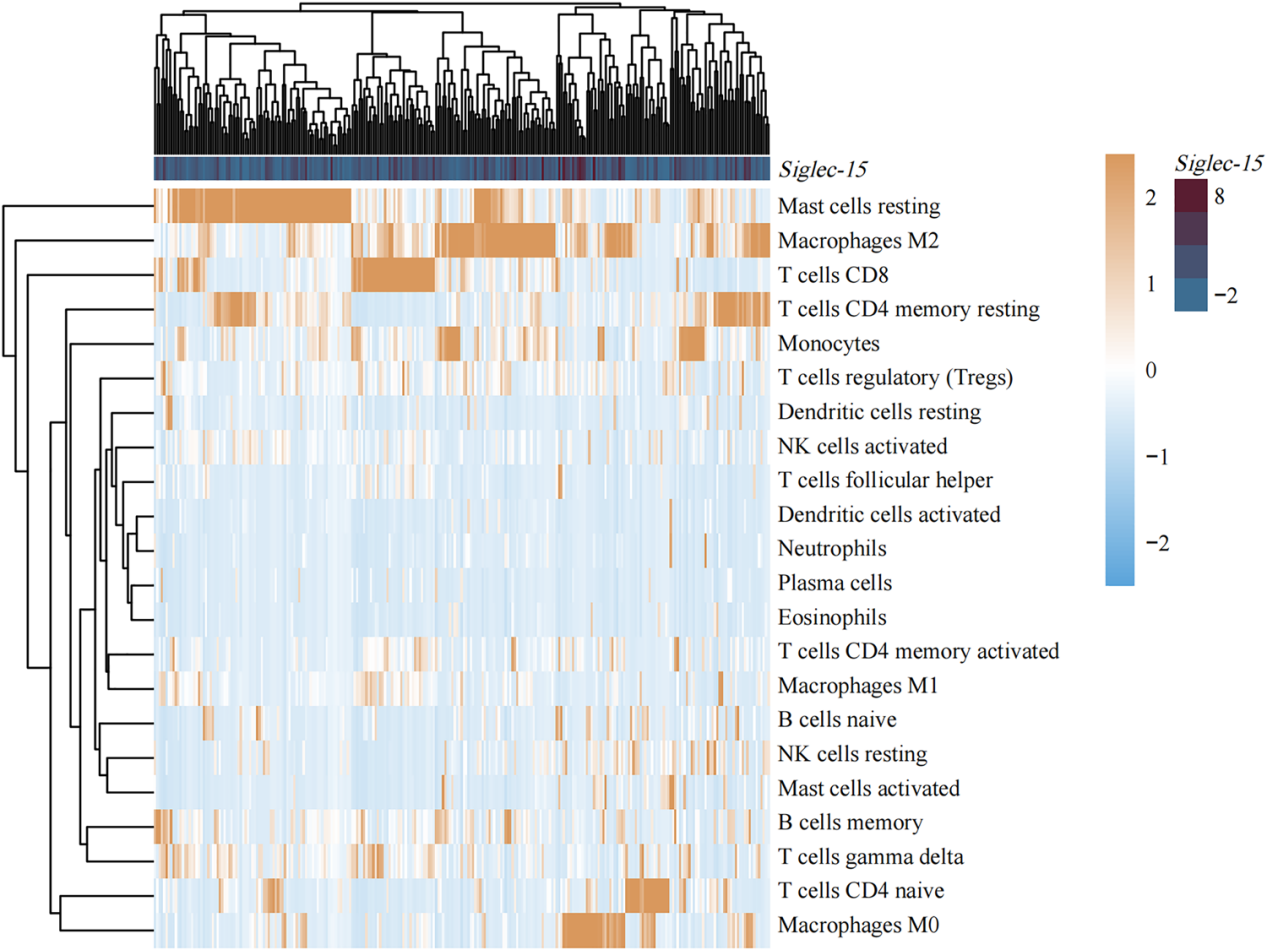
Heat map for correlation analysis between *Siglec-15* and immune cell subtypes.

After comprehensive analysis, we take *Siglec-15* as the research focus. Therefore, the correlation between *Siglec-15* and immune cell subtypes was further analyzed. Heat map showed that *Siglec-15* was significantly correlated with M2 macrophages (Fig. 11).

## Discussion

SARC are a highly heterogeneous cancer type comprising over 100 subtypes. Surgery and chemotherapy are the current mainstream treatments but have limited efficacy. Recently, cancer immunotherapy has led to impressive survival benefit for patients in some solid tumors, which, however, has made no similar breakthrough achievements in the treatment of sarcomas as seen in other malignancies^23,24^. Among immune checkpoint inhibitor therapies, PD-1/PD-L1 blockade has been investigated most often in sarcoma^25^. Tumor cells can exploit sialoglycan–Siglec interactions to modulate immune cell function, influencing the progression of tumor^26^. Several Siglec family members are expressed on sarcoma and associated with the prognosis of the patients.

In this study, we first examined the expression patterns of Siglec family members in SARC. The results showed that, compared with normal tissues, all members of the Siglec family were abnormally highly expressed in SARC. Based on the fact that the sample size of the normal SARC group in the TCGA database is small, we changed the analysis strategy. The gene sequencing data of SARC samples were downloaded, and divided into low expression group and high expression group with the median expression of each gene in the sample as the dividing point, and then the differences between the groups were compared. The results revealed that except for *Siglec-6*, *Siglec-8* and *Siglec-11*, most of the Siglec family members had inter-group differences. To investigate the prognostic value of each molecule, we performed a survival analysis. The results showed that low expression of *Siglec-6* and high expression of *Siglec-15* were associated with poor prognosis. These data fully demonstrate that the clinical prognostic value of the Siglec family in SARC cannot be ignored.

In order to understand the molecular features of the Siglec family more comprehensively, we systematically analyzed all the Siglec family molecules in SARC. The study found that, except for individual molecules, there were protein–protein interactions among most family members, and KEGG functional enrichment analysis showed that the signaling pathway was enriched in hematopoietic cell lineage. The above results indicate that the synergistic effect of the Siglec family may be related to the occurrence and development of SARC.

We found that genetic mutations were prevalent in Siglec family members in SARC patients, and mutants *Siglec-2* and *Siglec-15* were associated with poorer prognosis in sarcoma patients. Among these mutated genes, TP53 was the most mutated gene, followed by MUC16. TP53, a tumor suppressor gene, which was mutated in 50% of human tumors. The oncogenic function of mutated TP53 was very similar in SARC and various cancers, mainly in maintaining tumor cell proliferation and tumor growth^27^. Mutations in p53G245C and p53R273H have been reported to enhance the malignant potential of SARC cells, as well as their ability to proliferate and migrate^28–30^. All these molecular data suggested that TP53 might serve as a promising target for SARC therapy. MUC was a highly glycosylated protein with a single transmembrane structure that was secreted by epithelial cells and played a certain regulatory role in physiological and pathological processes such as signal transduction pathways and immune responses^31^. In particular, the impact on tumor had gradually attracted the attention of the academic community^32^. MUC16 was a member of the mucin family, and CA125 was encoded by the MUC16 gene^33^, which promoted cancer cell proliferation and inhibited anticancer immune responses. Studies have found that MUC16 can be mutated in a variety of SARC^34–36^, and targeting MUC16 may be a new breakthrough in the treatment of SARC. Based on the above information, *Siglec-15* has great potential as a breakthrough point in the treatment of sarcoma.

Furthermore, we assessed the immune-infiltrating signature of Siglecs in SARC based on the strong link between the Siglec family and the immune system. In our study, significant positive associations were found between all family members and multiple immune cells including B cells, CD8 + T cells, CD4 + T cells, macrophages, neutrophils and dendritic cells. Apart from that, we found a significant correlation between the somatic CNAs of the Siglec molecules and the abundance of immune infiltrates. *Siglec-15* as a potential gene, we further analyzed the association between *Siglec-15* and immune cell subtypes, and found that *Siglec-15* was significantly correlated with M2 macrophages. Taken together, members of the Siglec family not only were extensively involved in immune regulation, but may also predict response to immunotherapy and hold promise as new prognostic biomarkers.

Survival analysis showed that SARC patients with high expression of *Siglec-15* had poor prognosis. Therefore, we believe was that *Siglec-15* can independently predict patient survival. Song et al.^22^ performed IHC experiments on tumor tissue from SARC patients and found that *Siglec-15*-positive patients had significantly shorter OS than *Siglec-15*-negative patients (*p* = 0.015). Similarly, Fan et al.^37^ reported that *Siglec-15* was highly expressed in human osteosarcoma tissues, and the expression of *Siglec-15* was positively correlated with lung metastasis. Kaplan–Meier analysis showed that the high *Siglec-15* group had lower OS than the low *Siglec-15* group (*p* = 0.024). Taken together, the above results suggest that *Siglec-15* may be the most significant prognostic biomarker for SARC and may become a breakthrough point for SARC treatment.

This study has several limitations. First, our data analysis was only based on the TCGA database, and the data were relatively limited. Second, there was no more comprehensive and sufficient clinical trials or in vivo experiments to verify the reliability of the analysis results.

## Materials and methods

### GEPIA

Gene Expression Profiling Interactive Analysis (GEPIA, http://gepia.cancer-pku.cn/) is a tool online which is used to analyze the microarray data from TCGA datasets and GTEx projects^38^. We extracted the expression of each gene of Siglec family in different tumor types and SARC. We selected the “expression analysis” mode, entered Siglec family genes in the Gene list, and then selected “all” or “SARC” as cancer types. Other options were set to the default values.

### UALCAN

UALCAN (http://ualcan.path.uab.edu) is a user-friendly, interactive web resource to perform in-depth analyses of TCGA gene expression data^39^. This free portal is primarily used to analyze relative expression of a query genes across tumor and normal samples, explore the correlation between RNA expression and various tumor subtypes or some clinical characteristics such as individual age, gender, tumor stages. Therefore, we explored the relative expression of Siglec family molecules in tumor and normal samples of SARC via UALCAN. In the “TCGA gene analysis” module, the expression data of the Siglec family were analyzed using the “SARC” TCGA dataset. Statistical analysis was performed using a two-tailed Student t test, with a cutoff *p* value of < 0.05.

### OmicStudio

OmicStudio (https://www.omicstudio.cn/tool)^40^ is an integrated web tool for visualising user-defined data, including genomic, transcriptional (including single-cell analysis), and microbiomic information. Expression analysis of the Siglec family, as well as survival analysis, was performed using transcription data and clinical data of SARC downloaded from the TCGA database and visualized in OmicStudio.

### TIMER 2.0

The Tumor Immune Estimation Resource (TIMER) database version 2.0 (https://cistrome.shinyapps.io/timer/) provides an intuitive web interface for 6 major analytic modules including gene expression, clinical outcomes, somatic mutations, and somatic copy number alterations^41^. In this study, we estimated the prognostic value of Siglec and determined the independent prognostic predictors. In addition, the “gene” module was used to analyze the correlation between gene expression and immune infiltration of six immune cells (CD4 + T cells, CD8 + T cells, B cells, macrophages, neutrophils and dendritic cells) in SARC.

### cBioPortal

The cBio Cancer Genomics Portal (http://cbioportal.org) is an open-access resource for interactive exploration of multidimensional cancer genomics data sets, currently covering 282 cancer research^42^. In this study, the genetic mutation of Siglec family members and their correlation with patient survival in SARC were investigated using the cBioportal. All searches were performed according to the online instructions of cBioPortal.

### STRING

STRING (https://string-db.org/) is a database for analyzing and predicting protein functional connectivity and protein interactions, which includes direct (physical) and indirect (functional) associations^43^. It was used to generate the protein–protein interaction (PPI) network of associations for Siglec protein to better understand the functions and efficacy of Siglec. Enter all Siglec family members in the search box and select “Homo sapiens” as an organism. Other options were left as default options.

### GeneMANIA

GeneMANIA (http://www.genemania.org) is a flexible, user-friendly web interface for predicting functionally similar genes of hub genes and constructing the PPI network among them^44^. It can provide information for protein and genetic interactions, pathways, co-expression, co-localization, and protein domain similarity of submitted genes. GeneMANIA was used to analyze functionally similar genes of Siglec family members and perform functional enrichment analysis in this investigation.

### Statistical analysis

The Siglec expression levels between SARC tissue and normal tissue were evaluated by the GEPIA2 database. The survival analyses were estimated by GEPIA 2 and KM Plotter database, and the log-rank test was used to estimate the difference in survival rate. Furthermore, the correlation between Siglec expression and clinicopathological characteristics and immune cell infiltration was estimated by TIMER database. *P* value < 0.05 was considered as statistical significance.

## Data Availability

Publicly available datasets were analyzed in this study. This data can be found here: TCGA database (https://portal.gdc.cancer.gov/projects/TCGA-SARC).
